# Low-Frequency Raman Spectroscopy on Amorphous Poly(Ether Ether Ketone) (PEEK)

**DOI:** 10.3390/ma17153755

**Published:** 2024-07-30

**Authors:** Tomoko Numata, Naomoto Ishikawa, Toshihiro Shimada, Keith C. Gordon, Makoto Yamaguchi

**Affiliations:** 1 Department of Systems Design Engineering, Akita University, 1-1 Tegatagakuen-machi, Akita 010-8502, Japan; tomoko.numata@horiba.com; 2 Horiba Techno Service Co., Ltd., Chiyoda-ku, Tokyo 101-0063, Japan; 3 Kiguchi Technics Inc., 114-15 Enoshima-cho, Yasugi-shi 692-0057, Japan; ishikawa_n@kiguchitech.co.jp; 4 Division of Applied Chemistry, Faculty of Engineering, Hokkaido University, Kita 13 Nishi 8, Kita-ku, Sapporo 060-8628, Japan; shimadat@eng.hokudai.ac.jp; 5 Department of Chemistry, University of Otago, Dunedin 9016, New Zealand; keith.gordon@otago.ac.nz

**Keywords:** Raman spectroscopy, thermoplastic polymer, PEEK, CFRP, crystallinity, amorphous

## Abstract

Low-frequency peaks in the Raman spectra of amorphous poly(ether ether ketone) (PEEK) were investigated. An amorphous sample with zero crystallinity, as confirmed by wide-angle X-ray diffraction, was used in this study. In a previous study, two peaks were observed in the low-frequency Raman spectra of the crystallized samples. Among these, the peaks at 135 cm^−1^ disappeared for the amorphous sample. Meanwhile, for the first time, the peak at 50 cm^−1^ was observed in the crystallized sample. Similar to the peak at 135 cm^−1^, the peak at 50 cm^−1^ disappeared in the amorphous state, and its intensity increased with increasing crystallinity. The origins of the two peaks were associated with the Ph-CO-Ph-type intermolecular vibrational modes in the simulation. This suggests that the Ph-CO-Ph vibrational mode observed in the low-frequency region of PEEK was strongly influenced by the intermolecular order.

## 1. Introduction

**Figure 1:**
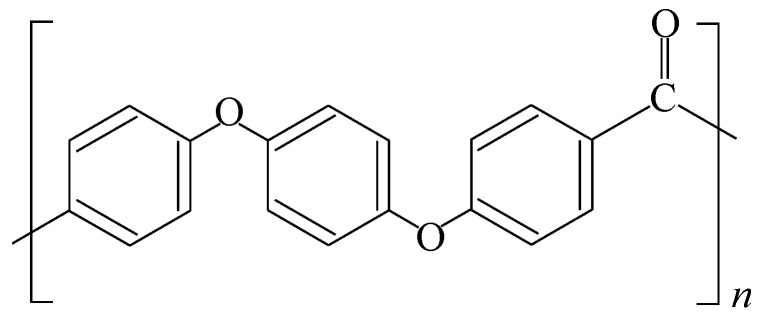
Chemical structure of PEEK.

In recent years, carbon fiber–reinforced plastic (CFRP) has gained attention in multiple fields, including aircraft and automobile fuselage materials, due to its potential as an alternative to conventional steel materials. CFRP offers equivalent or superior strength and a substantial weight reduction, making it an attractive option. There are two primary types of CFRP: thermosetting CFRP, which uses a thermosetting polymer as the base material, and thermoplastic CFRP, which uses a thermoplastic polymer as the matrix material. Thermoplastic polymers soften and melt when heated and solidify when cooled. Thermoplastic CFRP has attracted attention in various fields because it requires a short molding time, is inexpensive, and has high productivity [1]. Poly(ether ether ketone) (PEEK) (Figure 1) is a high-performance thermoplastic polymer developed by Imperial Chemical Industries (now Victrex) in Thornton-Cleveleys, UK, and it has excellent mechanical and chemical properties and thermal stability [2]. The degree of crystallinity of the crystalline polymers greatly influences their mechanical, optical, electrical, and other properties [3,4,5,6,7,8,9,10,11,12]. As such, the evaluation of the crystallinity of PEEK is highly important [13,14]. 

Numerous techniques have been utilized to evaluate the crystallinity of polymers, including PEEK [14]. Among these, Wide-angle X-ray diffraction (WAXRD), differential scanning calorimetry (DSC) [15], and Fourier-transform infrared spectroscopy [13] are frequently used techniques. In composite materials, the crystal structure of polymers and behavior of crystal nucleus precipitation are reported to differ near fibers [16,17]. The degree of crystallinity is expected to be spatially unevenly distributed owing to the influence of the carbon fibers. Therefore, a crystallinity evaluation method with a high spatial resolution is required.

Raman spectroscopy is one of the most commonly used techniques for investigating polymer material [18]. In addition, high spatial resolution can be achieved using a microscopic optical system. Previous studies used Raman spectroscopy to assess the crystallinity of PEEK [14,19,20,21,22]. As carbon materials exhibit prominent D- and G-band signals at approximately 1600 cm^−1^ [23], interference between the signals from carbon and polymer is a problem for composite materials. Consequently, frequencies within a range without carbon signals should be employed. To address this problem, we recently reported the evaluation of the crystallinity of PEEK using the peak in the low-frequency region (<100 cm^−1^) [24].

Raman scattering in the low-frequency region has attracted attention in various fields because it is easier to measure owing to the development of optical components. Similarly, remarkable progress has been made in the research of spectroscopy in the terahertz region in recent years. In some polymer materials, the peaks obtained by low-frequency Raman scattering spectroscopy [25,26,27,28] and THz vibrational spectroscopy [29,30,31] are caused by intermolecular vibrations. Classical molecular dynamics (MD) simulations have noted the intermolecular vibrational mode in the terahertz region for n-poly-l-lactide [32]. For PEEK, intermolecular ordering affects the low-frequency Raman spectra, as obtained by MD simulations [33]; however, no direct experimental evidence has been reported. We investigated the behavior of low-frequency Raman spectra in an amorphous sample, which is thought to have no intermolecular ordering.

The origins of low-frequency peaks, which are reliable indicators of crystallinity, should be clarified. This study aims to evaluate the Raman spectra of amorphous PEEK in the low-frequency region using a sample with zero crystallinity and discuss the origin of these peaks.

## 2. Materials and Methods

### 2.1. Sample Preparation

A Grade 2000 APTIV PEEK film produced by Victrex (t = 25 μm, UK), which is an unfilled amorphous film with zero crystallinity [14], was used as the sample. Various degrees of crystallinity were achieved by the heat treatment of the as-received films. Heat treatment above the glass transition temperature and below the melting temperature induces cold crystallization, resulting in an increased degree of crystallinity. The heat-treatment temperatures of 150, 175, 200, 250, and 300 °C were selected. The samples were kept at the annealing temperature for 2 h in air before they were cooled to room temperature in the same environment.

### 2.2. Experiments

WAXRD (Rigaku Ultima IV, Tokyo, Japan) was employed at room temperature to determine crystallinity. CuKα X-rays with a wavelength of 0.154 nm were used, with a rotating anode operating at 40 kV and 30 mA. The diffraction angle, 2θ, was in the range from 5° to 40°. 

A micro-Raman spectroscopic system (Horiba Jobin Yvon, LablamHR spectrometer, Kyoto, Japan) with a backscattered geometry was used. The spot diameter was approximately 1 μm using an objective lens of 50×. In our PEEK sample, highly strong fluorescence was observed when excited at 633 nm or 532 nm, which are commonly used wavelengths in Raman scattering spectroscopy. An infrared light with a wavelength of 1064 nm was selected as the excitation light to avoid fluorescence. In order to measure the low-frequency region, a notch filter capable of measuring up to 20 cm^−1^ (the ultra-low-frequency (ULF) module) was used. An InGaAs detector with a resolution of 1.83 cm^−1^/pixel using cooled liquid nitrogen was used. The laser power incident on the sample was limited to 60 mW to avoid damage and further crystallization. The exposure duration was 10 s, and the measurements were integrated four times.

### 2.3. Density Functional Theory (DFT) and MD Simulation

The molecular vibrations of single-stranded PEEK oligomers were calculated using DFT (B3LYP/6-31G) in Gaussian16. The vibrations of the crystalline PEEK were calculated using MD simulations in a large-scale atomic/molecular massively parallel simulator (LAMMPS, 2 Aug 2023 version) with a generalized AMBER FF (GAFF, 2004 version). The calculation details are provided in Ref. [31].

## 3. Results and Discussion

### 3.1. WAXRD Diffractograms

**Figure 2:**
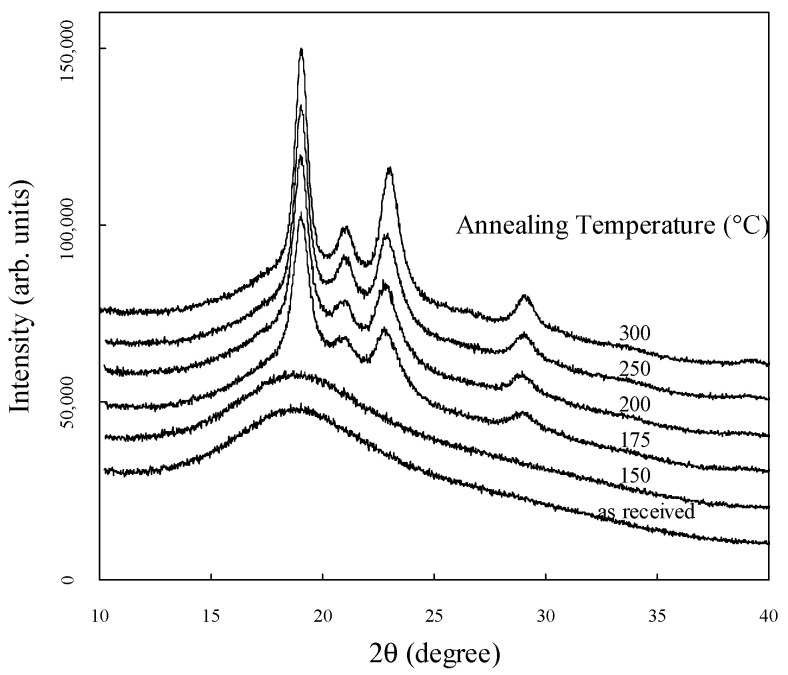
WAXRD diffractograms measured from 5° to 40° for different temperatures.

Figure 2 shows the WAXRD diffractograms measured from 5° to 40° for each annealing temperature. The diffractograms are shown on the same scale and are vertically shifted to show them more clearly. Sharp peaks are observed at 2θ values of approximately 19°, 21°, 23°, and 29°, corresponding to the 110, 111, 200, and 211 planes, respectively [34]. The broad peak observed at approximately 19° is associated with the amorphous structure. In the as-received sample without annealing, no crystallinity peak is observed, similar to previous studies [14,35]. The crystallinity was determined by fitting each obtained WAXRD spectrum to separate sharp peaks due to crystallinity and broad peaks due to the amorphous structure of the Lorentzian and Gaussian functions, respectively.

### 3.2. Raman Spectra

**Figure 3:**
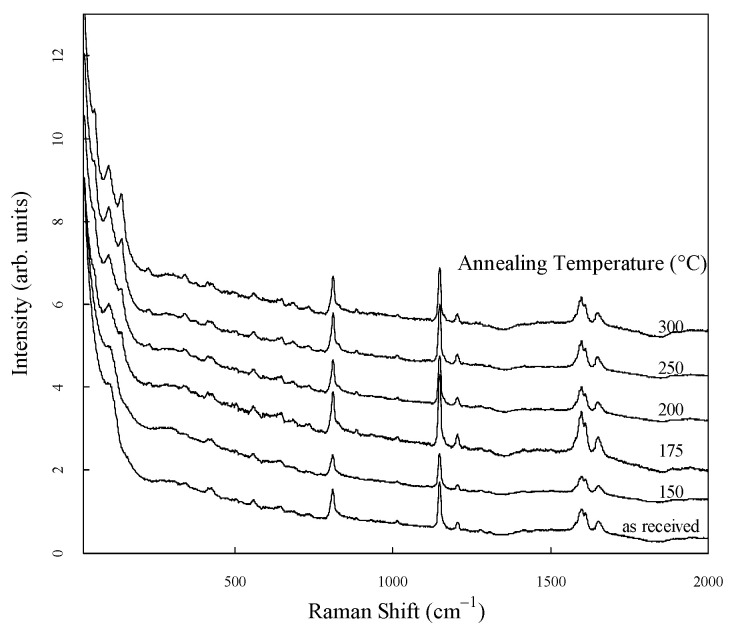
Standardized Raman spectra of PEEK in the range of 40 cm^−1^ to 2000 cm^−1^.

The Raman spectra of PEEK, standardized in the range of 40 cm^−1^ to 2000 cm^−1^, are shown in Figure 3. For clarity, the spectra are shown to be vertically shifted. Several strong peaks are observed in the low- and mid-frequency regions of all spectra. The origins of the peaks in the mid-frequency region have been identified in previous studies [19,20,21,22] and have been summarized in a recent report [14].

**Figure 4:**
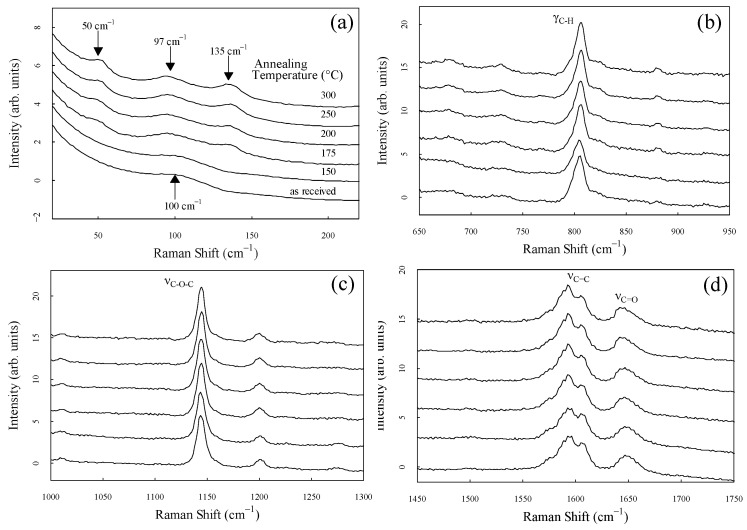
Enlarged spectra in the frequency range of (**a**) 20–220 cm^−1^, (**b**) 650–950 cm^−1^, (**c**) 1000–1300 cm^−1^, and (**d**) 1450–1750 cm^−1^. Each spectrum was standardized along the horizontal axis.

Enlarged spectra of each region are shown in Figure 4a–d to observe the spectral shape in more detail. Each spectrum was standardized in the area displayed on the horizontal axis. Figure 4a shows the peaks at 50 cm^−1^, 97 cm^−1^, and 135 cm^−1^ in samples annealed at temperatures above 175 °C. In our previous study, we reported that the peak at 135 cm^−1^ was significantly related to crystallinity and associated with intermolecular vibration modes [24,33]. The peak at 50 cm^−1^, which is markedly dependent on the degree of crystallinity, similar to the peak at 135 cm^−1^, is observed for the first time. Crystallinity dependence has been reported for various peaks in the mid-frequency region, such as the peak center at 1644 cm^−1^, intensity ratio of 1597 cm^−1^ and 1607 cm^−1^, intensity ratio of the bands at 800 cm^−1^ and 810 cm^−1^ [19], and center frequency at 1146 cm^−1^ [36] and 1651 cm^−1^ [14]. Similarly, the peaks at 50 cm^−1^ and 135 cm^−1^ in the low-frequency region are highly sensitive to crystallinity. Furthermore, the peaks at 50 cm^−1^ and 135 cm^−1^ completely disappeared in the sample with zero crystallinity.

**Figure 5:**
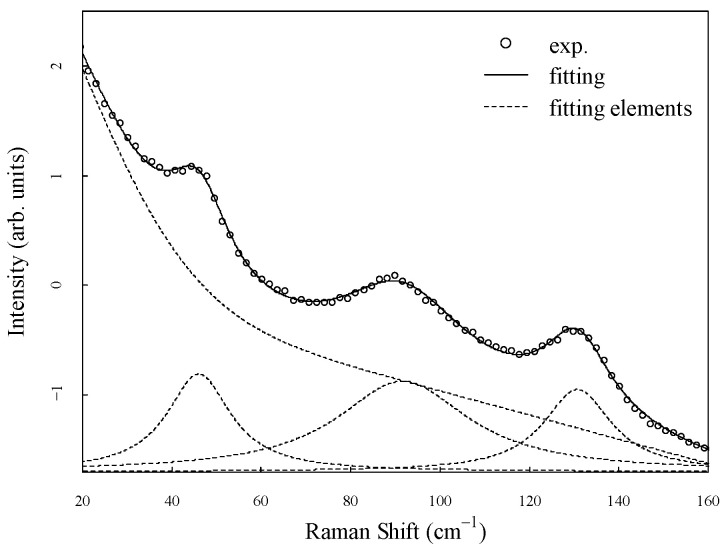
A typical example of the fitting analysis of the Raman spectra. The dashed lines represent the decomposed elements with three peaks and a baseline curve.

The Raman spectra in the low-frequency region were decomposed into peaks at 50 cm^−1^, 97 cm^−1^, and 135 cm^−1^ expressed by a Lorenz function, a Gaussian function, and a linear function as the baseline data. The Lorenz function can be characterized based on the intensity, half-width at half maximum, and center frequency. The Gaussian function represents the background with a center frequency of 0 cm^−1^, and the half-width at half maximum and intensity are used as fitting parameters. Each parameter was independently adjusted to achieve the minimum chi-square value using the Levenberg-Marquardt nonlinear least-squares optimization. A sample fit of the Raman spectra is shown in Figure 5.

**Figure 6:**
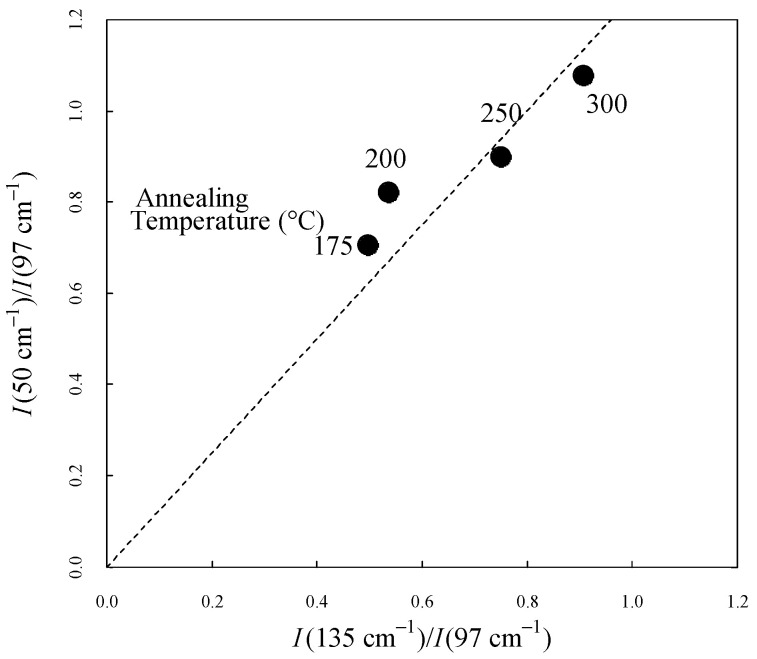
Correlation between $I_{50{cm}^{-1}}/I_{135\;{cm}^{-1}}$ and $I_{50{cm}^{-1}}/I_{97{cm}^{-1}}$. The dashed line represents a visual guide.

Figure 6 shows the intensity ratio between 50 cm^−1^ and 97 cm^−1^ ($I_{50{cm}^{-1}}/I_{97{cm}^{-1}}$) and between 135 cm^−1^ and 97 cm^−1^ ($I_{135{cm}^{-1}}/I_{97{cm}^{-1}}$). The dashed line demonstrates the good correlation of the parameters. Both values increased with increasing crystallinity. Along with the disappearance of the peaks in the amorphous state, this result suggests that the two peaks were influenced by the intermolecular order through the same mechanism.

### 3.3. DFT and MD Simulations

**Figure 7:**
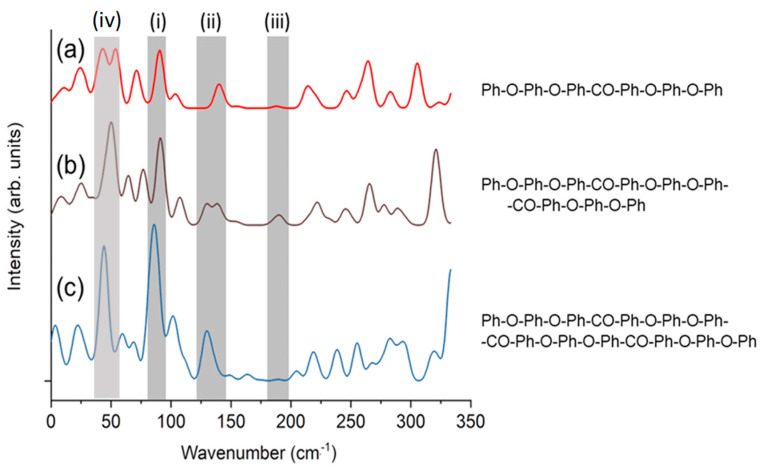
Raman spectra of phenyl-terminated PEEK oligomers calculated using DFT. The peaks at 85–98 cm^−1^, 130–145 cm^−1^, 190 cm^−1^, and 50 cm^−1^ are labeled as (i), (ii), (iii), and (iv), respectively. (**a**–**c**) represent different lengths of a PEEK molecular chain. The molecular structures are shown in the right panel. The calculated molecular structures are shown on the right, where “Ph-”, “-Ph-”, “-O-”, and “-CO-” represent C6H5-, -C6H4-, ether, and ketone, respectively.

**Figure 8:**
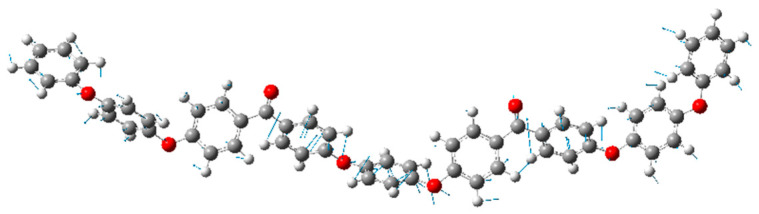
Atomic motion in the vibrational modes at 50 cm^−1^ calculated for a single oligomer molecule using DFT. The white, red, and gray spheres represent hydrogen, oxygen, and carbon, respectively. The blue lines show the direction of the movement of the atom, and their lengths show the magnitude of the displacement.

Simulations were performed to clarify the origin of the peak at 50 cm^−1^. Figure 7 shows the Raman spectra calculated using DFT. Figure 7, which was modified from previous work Ref. [28], shows the spectra of PEEK oligomers with different lengths, terminated with phenyl groups. Peaks (i)–(iii) in the gray-shaded region are described in previous work [33]. Figure 8 shows the atomic motion in the vibrational modes at approximately 50 cm^−1^ (peak (iv)) calculated for a single oligomer molecule using DFT. The peak at 50 cm^−1^ is ascribed to the twisting motion of the ketone and connected benzene rings (Ph-O-Ph), similar to the peak at 135 cm^−1^ (peak (ii)). MD simulations were performed using LAMMPS. The simulation video is provided as Supplemental Video S1, whereby the time interval corresponds to 50 cm^−1^. The peaks at 50 cm^−1^ and 135 cm^−1^ are associated with Ph-CO-Ph-type intermolecular vibrational modes. Ketone groups have high polarity; therefore, the ketone group plays a major role in molecular interactions. Moreover, the peaks at 50 cm^−1^ and 135 cm^−1^ are not observed in the amorphous state and are sensitive to crystallinity. They are related to ketone groups, which are strongly related to the intermolecular order.

Delbé et al. observed three peaks at 77 cm^−1^, 109 cm^−1^, and 135 cm^−1^ in the low-frequency region of poly(ether ketone ketone) (PEKK) [37]. The peaks at 109 and 135 cm^−1^ in PEKK corresponded to the peaks at 97 and 135 cm^−1^ in PEEK, respectively. The vibration frequency decreased because of the addition of a ketone group in PEKK. Thus, the peak at 50 cm^−1^ in PEEK corresponds to the peak at 77 cm^−1^ in PEKK (Table 1). Among the three peaks in the low-frequency region observed for crystalline PEEK, two peaks assigned to ketone group vibrations disappeared in the amorphous PEEK. In contrast, a peak assigned to the vibration of the ketone group is observed even in the amorphous state. The Ph-CO-Ph vibration mode was enhanced by intermolecular interactions in PEEK and intramolecular interactions in PEKK. In the future, further clarification on this matter will be provided through extensive simulations focused on ketone groups.

## 4. Conclusions

The low-frequency peaks in the Raman spectra of the amorphous PEEK were studied. An amorphous sample with zero crystallinity, as confirmed by WAXRD, was used in this study. The peaks at 50 cm^−1^, which were observed for the first time, and 135 cm^−1^ disappeared in the amorphous sample. The peak intensities at 135 cm^−1^ and 50 cm^−1^ increased as the crystallinity increased. Along with the disappearance of the peaks in the amorphous state, these results suggest that the two peaks were influenced by the intermolecular order through the same mechanism. The peak at 50 cm^−1^ indicates the twisting motion of the ketone and connected benzene rings (Ph-O-Ph), which were similar to the peak at 135 cm^−1^ obtained using DFT and MD simulations. Furthermore, the peaks in the low-frequency region of PEEK and PEKK suggested the important role of the ketone group.

## Figures and Tables

**Table 1:** Raman peak of PEEK and PEKK [37] in the low-frequency region.

PEEK		PEKK	Assignment
-Ph-O-Ph-O-Ph-CO-		-Ph-O-Ph-CO-Ph-CO-	
crystal	amorphous	amorphous	
wavenumber (cm^−1^)		wavenumber (cm^−1^)	
50	Not observed	77	phonon Ph-CO-Ph
97	100	109	phonon Ph-O-Ph
135	Not observed	135	phonon Ph-CO-Ph

## Data Availability

The original contributions presented in the study are included in the article/supplementary material, further inquiries can be directed to the corresponding author.

## Supplementary Materials

Video S1: MD snapshots corresponding to vibration at 50 cm^−1^. Available online: https://youtu.be/sS23bM8gP8g (accessed on 30 May 2024).
